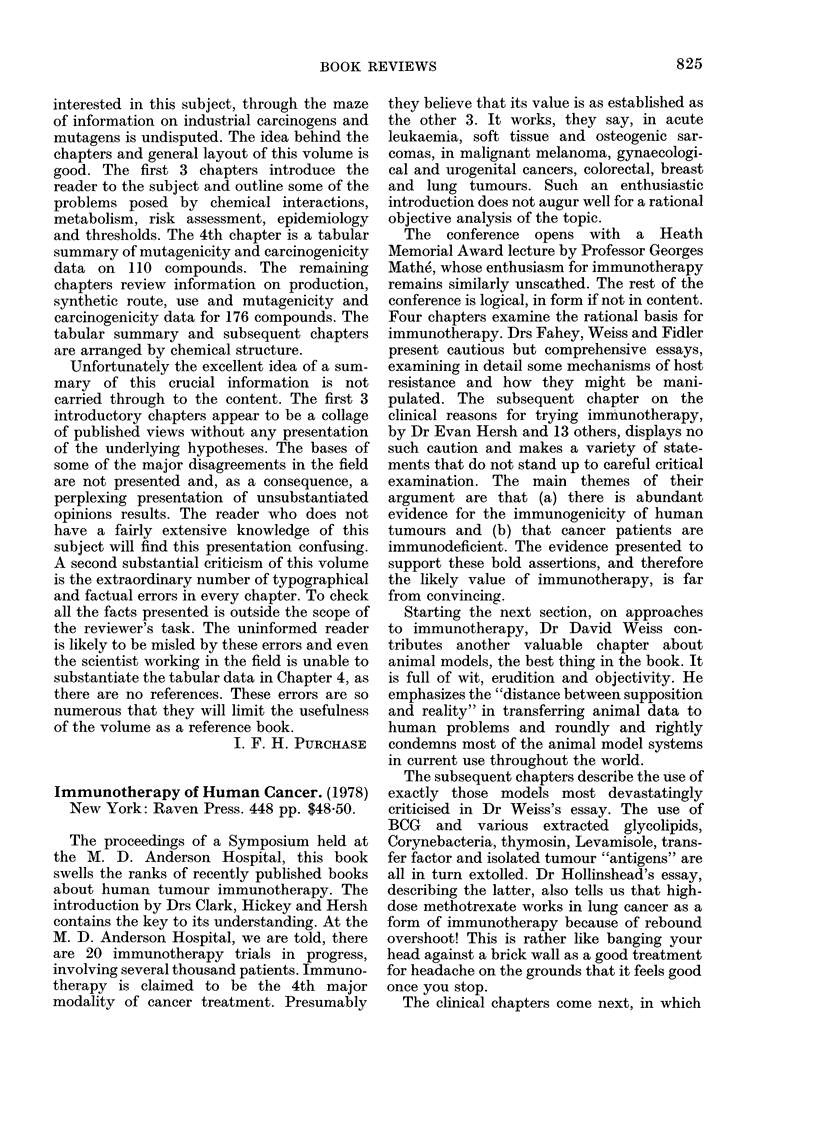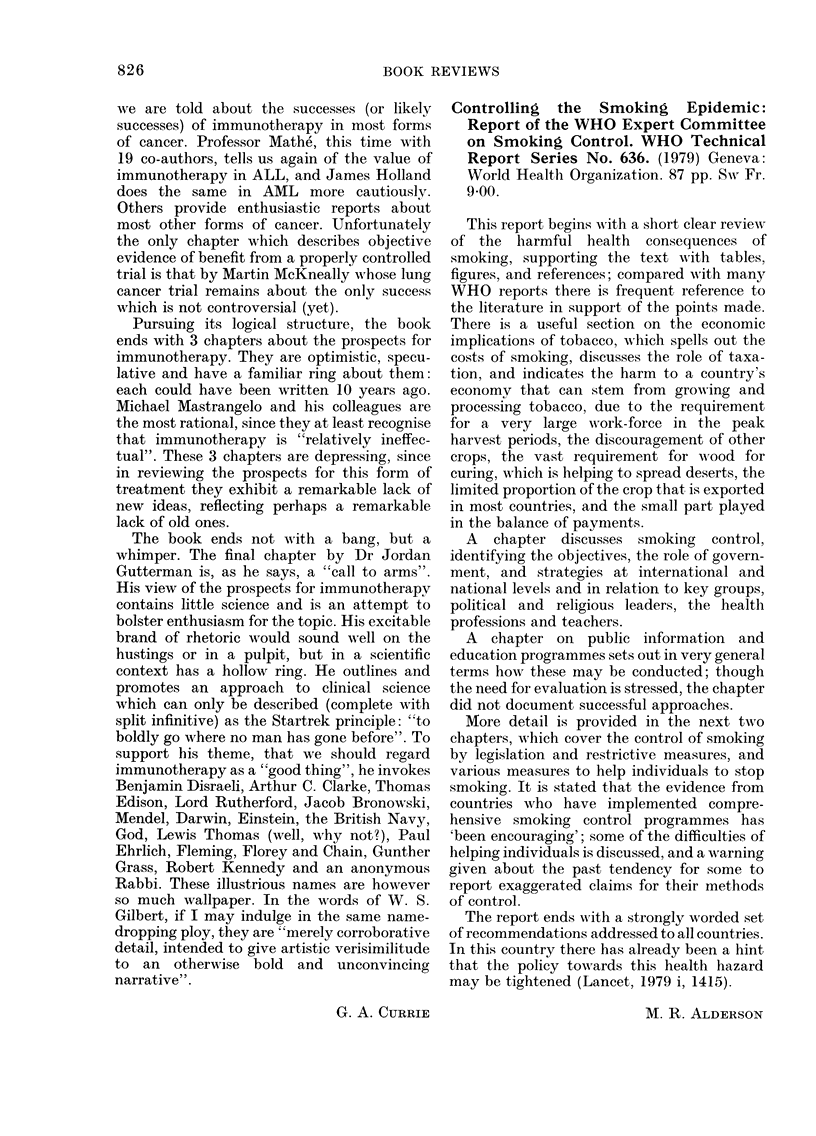# Immunotherapy of Human Cancer

**Published:** 1979-11

**Authors:** G. A. Currie


**Immunotherapy of Human Cancer.** (1978) New York: Raven Press. 448 pp. $48·50.

The proceedings of a Symposium held at the M. D. Anderson Hospital, this book swells the ranks of recently published books about human tumour immunotherapy. The introduction by Drs Clark, Hickey and Hersh contains the key to its understanding. At the M. D. Anderson Hospital, we are told, there are 20 immunotherapy trials in progress, involving several thousand patients. Immunotherapy is claimed to be the 4th major modality of cancer treatment. Presumably they believe that its value is as established as the other 3. It works, they say, in acute leukaemia, soft tissue and osteogenic sarcomas, in malignant melanoma, gynaecological and urogenital cancers, colorectal, breast and lung tumours. Such an enthusiastic introduction does not augur well for a rational objective analysis of the topic.

The conference opens with a Heath Memorial Award lecture by Professor Georges Mathé, whose enthusiasm for immunotherapy remains similarly unscathed. The rest of the conference is logical, in form if not in content. Four chapters examine the rational basis for immunotherapy. Drs Fahey, Weiss and Fidler present cautious but comprehensive essays, examining in detail some mechanisms of host resistance and how they might be manipulated. The subsequent chapter on the clinical reasons for trying immunotherapy, by Dr Evan Hersh and 13 others, displays no such caution and makes a variety of statements that do not stand up to careful critical examination. The main themes of their argument are that (a) there is abundant evidence for the immunogenicity of human tumours and (b) that cancer patients are immunodeficient. The evidence presented to support these bold assertions, and therefore the likely value of immunotherapy, is far from convincing.

Starting the next section, on approaches to immunotherapy, Dr David Weiss contributes another valuable chapter about animal models, the best thing in the book. It is full of wit, erudition and objectivity. He emphasizes the "distance between supposition and reality" in transferring animal data to human problems and roundly and rightly condemns most of the animal model systems in current use throughout the world.

The subsequent chapters describe the use of exactly those models most devastatingly criticised in Dr Weiss's essay. The use of BCG and various extracted glycolipids, Corynebacteria, thymosin, Levamisole, transfer factor and isolated tumour "antigens" are all in turn extolled. Dr Hollinshead's essay, describing the latter, also tells us that high-dose methotrexate works in lung cancer as a form of immunotherapy because of rebound overshoot! This is rather like banging your head against a brick wall as a good treatment for headache on the grounds that it feels good once you stop.

The clinical chapters come next, in which we are told about the successes (or likely successes) of immunotherapy in most forms of cancer. Professor Mathé, this time with 19 co-authors, tells us again of the value of immunotherapy in ALL, and James Holland does the same in AML more cautiously. Others provide enthusiastic reports about most other forms of cancer. Unfortunately the only chapter which describes objective evidence of benefit from a properly controlled trial is that by Martin McKneally whose lung cancer trial remains about the only success which is not controversial (yet).

Pursuing its logical structure, the book ends with 3 chapters about the prospects for immunotherapy. They are optimistic, speculative and have a familiar ring about them: each could have been written 10 years ago. Michael Mastrangelo and his colleagues are the most rational, since they at least recognise that immunotherapy is "relatively ineffectual". These 3 chapters are depressing, since in reviewing the prospects for this form of treatment they exhibit a remarkable lack of new ideas, reflecting perhaps a remarkable lack of old ones.

The book ends not with a bang, but a whimper. The final chapter by Dr Jordan Gutterman is, as he says, a "call to arms". His view of the prospects for immunotherapy contains little science and is an attempt to bolster enthusiasm for the topic. His excitable brand of rhetoric would sound well on the hustings or in a pulpit, but in a scientific context has a hollow ring. He outlines and promotes an approach to clinical science which can only be described (complete with split infinitive) as the Startrek principle: "to boldly go where no man has gone before". To support his theme, that we should regard immunotherapy as a "good thing", he invokes Benjamin Disraeli, Arthur C. Clarke, Thomas Edison, Lord Rutherford, Jacob Bronowski, Mendel, Darwin, Einstein, the British Navy, God, Lewis Thomas (well, why not?), Paul Ehrlich, Fleming, Florey and Chain, Gunther Grass, Robert Kennedy and an anonymous Rabbi. These illustrious names are however so much wallpaper. In the words of W. S. Gilbert, if I may indulge in the same name-dropping ploy, they are "merely corroborative detail, intended to give artistic verisimilitude to an otherwise bold and unconvincing narrative".

G. A. Currie